# Functional characterization and target discovery of glycoside hydrolases from the digestome of the lower termite *Coptotermes gestroi*

**DOI:** 10.1186/1754-6834-4-50

**Published:** 2011-11-14

**Authors:** João Paulo L Franco Cairo, Flávia C Leonardo, Thabata M Alvarez, Daniela A Ribeiro, Fernanda Büchli, Ana M Costa-Leonardo, Marcelo F Carazzolle, Fernando F Costa, Adriana F Paes Leme, Gonçalo AG Pereira, Fabio M Squina

**Affiliations:** 1 Laboratório Nacional de Cência e Tecnologia do Bioetanol (CTBE), Centro Nacional de Pesquisa em Energia e Materiais (CNPEM), Campinas, Brazil; 2 Genomic and Expression Laboratory (LGE), Genetic, Evolution and Bioagents Department, State University of Campinas, Campinas, Brazil; 3 Hematology and Hemotherapy Center, University of Campinas, Campinas, Brazil; 4 Departamento de Biologia, Instituto de Biocências, Universidade Estadual Paulista (UNESP), Rio Claro, Brazil; 5 Laboratório Nacional de Biociencias (LNBio), Centro Nacional de Pesquisa em Energia e Materiais (CNPEM), Campinas, Brazil

## Abstract

**Background:**

Lignocellulosic materials have been moved towards the forefront of the biofuel industry as a sustainable resource. However, saccharification and the production of bioproducts derived from plant cell wall biomass are complex and lengthy processes. The understanding of termite gut biology and feeding strategies may improve the current state of biomass conversion technology and bioproduct production.

**Results:**

The study herein shows comprehensive functional characterization of crude body extracts from *Coptotermes gestroi *along with global proteomic analysis of the termite's digestome, targeting the identification of glycoside hydrolases and accessory proteins responsible for plant biomass conversion. The crude protein extract from *C. gestroi *was enzymatically efficient over a broad pH range on a series of natural polysaccharides, formed by glucose-, xylose-, mannan- and/or arabinose-containing polymers, linked by various types of glycosidic bonds, as well as ramification types. Our proteomic approach successfully identified a large number of relevant polypeptides in the *C. gestroi *digestome. A total of 55 different proteins were identified and classified into 29 CAZy families. Based on the total number of peptides identified, the majority of components found in the *C. gestroi *digestome were cellulose-degrading enzymes. Xylanolytic enzymes, mannan- hydrolytic enzymes, pectinases and starch-degrading and debranching enzymes were also identified. Our strategy enabled validation of liquid chromatography with tandem mass spectrometry recognized proteins, by enzymatic functional assays and by following the degradation products of specific 8-amino-1,3,6-pyrenetrisulfonic acid labeled oligosaccharides through capillary zone electrophoresis.

**Conclusions:**

Here we describe the first global study on the enzymatic repertoire involved in plant polysaccharide degradation by the lower termite *C. gestroi*. The biochemical characterization of whole body termite extracts evidenced their ability to cleave all types of glycosidic bonds present in plant polysaccharides. The comprehensive proteomic analysis, revealed a complete collection of hydrolytic enzymes including cellulases (GH1, GH3, GH5, GH7, GH9 and CBM 6), hemicellulases (GH2, GH10, GH11, GH16, GH43 and CBM 27) and pectinases (GH28 and GH29).

## Background

Fossil fuels are the world's primary energy source and a major issue regarding greenhouse gases emission. The key mitigation action is replacing petroleum and its derivatives with renewable energy sources [1]. In this context, lignocellulosic materials, which are an abundant and low-cost source of stored energy in the biosphere, have been moved towards the forefront of the biofuel industry as a sustainable resource [2]. However, saccharification and production of bioproducts derived from plant cell walls are complex and usually lengthy processes. For example, cellulosic biomass must go through an intensive pretreatment step, in which enzymes are used to break down biomass into simple sugars suitable for fermentation and bioethanol production. The protein repertoire involved in biomass conversion includes glycoside hydrolases (GHs), proteins with carbohydrate-binding modules (CBMs), glycoside transferases (GTs), laccases, peroxidases and detoxification proteins, such as superoxide dismutase and catalases [3]. These proteins are normally produced by fungi, bacteria, protozoa and even by animals [4], such as worms, beetles, cockroaches and termites, that carry complex symbiotic systems with cellulolytic microorganisms in their guts [5].

Termites are considered the smallest and most efficient decomposing bioreactors of wood on earth [6]. Within one microliter of gut environment, it is possible for them to break down lignocellulosic material into monosaccharides with 90% efficiency [7]. One of the best ways to understand termite digestomes is by coupling classical biochemical characterization with genomic and proteomic tools [8,9] based on a metagenomic platform for bioprospecting novel glycoside hydrolases [10].

In the last five years, a series of efforts on metagenomics and metatranscriptomics from termites were reported, discovering genes encoding GHs, GTs and CBMs [11-14], as well as components involved in the oxidation or detoxification of lignin [15,16]. Thus, these sets of genes, enzymes, proteins and co-factors produced by the termite and its symbionts (bacteria, yeast and protozoa) were named as the 'digestome' [11]. Warnecke *et al*. [14] identified 700 genes encoding GH and accessory proteins (mainly from bacteria) in a metagenomic library from the higher termite *Nasutitermes sp.*, as well as 13 enzymes identified by liquid chromatography with tandem mass spectrometry (LC-MS/MS) from its digestome. Burnum *et al*. [17] identified 886 proteins, including 22 polysaccharide hydrolases, from the proteome of the higher termite *N. corniger*. This proteomic effort enabled the reconstruction of metabolic pathways involved in termite symbiotic relationships.

*C. gestroi *is classified as a lower termite, belonging to the Rhinotermitidae family [18], which was introduced to Brazil in the early years of the past century and, at the present time, is regarded as the most important urban and building pest in the country. Gene expression patterns related to social cast differentiation, metabolic pathways and cellulose degrading enzymes, such as endo-β-1,4-glucosidase and β-1,4-glucosidase, were identified by transcriptome analysis from its head [19].

The goal of this work was to characterize the digestome of the lower termite *C. Gestroi*, with the aim of discoving novel carbohydrate-active enzymes. Our strategy consisted of classical biochemical characterization followed by global proteomic analysis of the termite digestome, targeting the identification of GHs and accessory proteins responsible for plant biomass degradation. The understanding of termite gut biology and feeding strategies may improve the current state of technology on biomass conversion and bioproduct production, which are still far away from economic efficiency.

## Results

### Biochemical characterization

**Figure 1:**
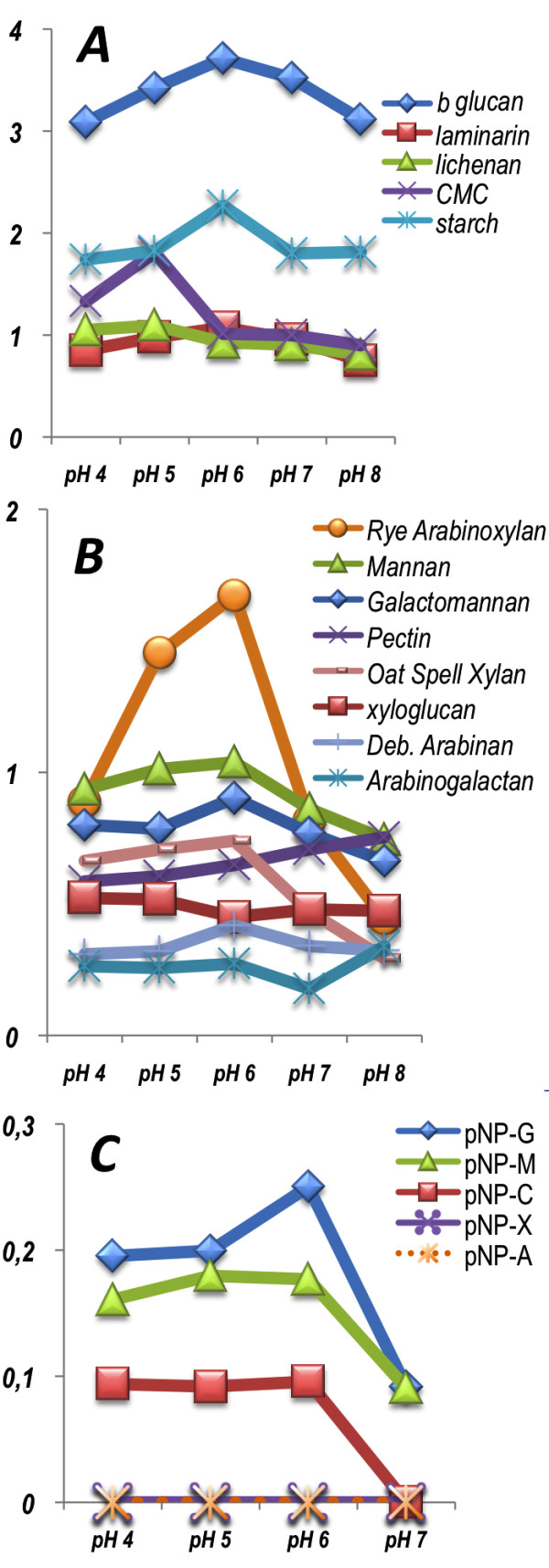
**Enzymatic activity evaluation of termite crude extract against commercial polysaccharides and synthetic substrates at different pHs**. **(A) **Enzymatic assays using glucose-containing polysaccharides. **(B) **Enzymatic assays using hemicellulosic polysaccharides. **(C) **Enzymatic assays using p-nitrophenyl derivatives.

Here we describe the first global study of an enzymatic repertoire involved in plant polysaccharide degradation by *C. gestroi*. The crude extract was highly active on hydrolyzing glucose-containing polysaccharides, in both the linear and branched forms (Figure 1A), such as β-glucan from barley; lichenan, a β-1-3:1-4 glucose linked polysaccharide; and laminarin, a β-1-3:1-6 glucose linkage polysaccharide. On these substrates, enzyme activity was over 70% active on a wide range of pH, from pH 4 to pH 8 (Figure 1A). The crude enzyme extract showed activity on carboxymethyl cellulose (CMC), a linear β-(1,4) polymer of D-glucose (Figure 1A). The relative enzymatic activity on CMC decreased to 73% at pH 4 and about 50% when incubated on pH 7 and pH 8. The enzymatic activity was also detected on starch, which is an α-linked glucose polysaccharide, and retained more than 77% of its enzymatic performance at pHs ranging from pH 4 to pH 8 (Figure 1A).

The termite crude extract was also competent in hydrolyzing hemicellulosic substrates (Figure 1B), such as mannan and galactomannan, as well as xylan from birch wood, a decorated β-1-4 xylose-containing polysaccharide. The enzymatic activity was also observed on xyloglucan and arabinoxylan, two β-1-4 glucose- and β-1-4 xylose-containing polymers, with lateral sugar branches of xylose and arabinose respectively. The enzymatic hydrolysis of pectin, which is composed of 1,4-linked α-D-galactosyluronic acid residues, was evidenced as well. Relatively low activities were observed over arabinogalactan and debranched arabinan. The optimum pH of operation in most of these substrates was between pH 5 and pH 6; nevertheless, more than 80% of activity at pH 4 and pH 8 was retained (Figure 1B).

**Figure 2:**
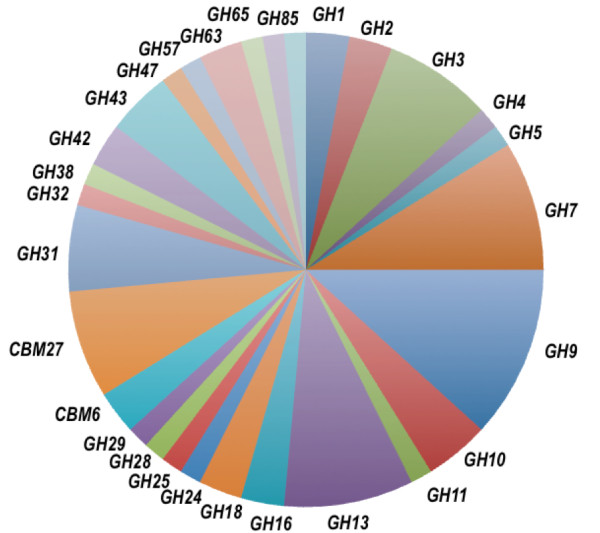
**Distribution of different unique peptides identified with similarity with CAZy families**. MS/MS analyses from *C. gestroi *digestome showed unambiguous assignments for 68 different unique peptides, which were distributed in 29 different CAZy families. MS/MS: in tandem mass spectrometry.

**Figure 3:**
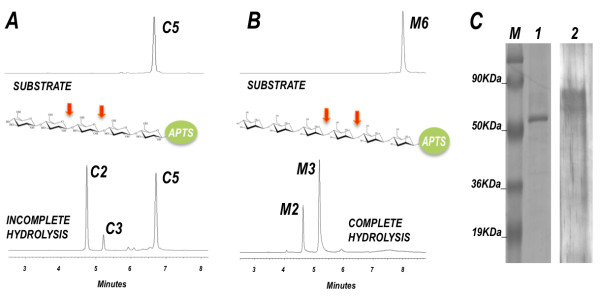
**Capillary zone electrophoresis of APTS-labeled oligosaccharides and SDS-PAGE analysis of FPLC positive screened derived fraction**. **(A) **Enzymatic activity mediated by IEX4-37 on cellopentaose, a positively screened FPLC fraction derived from *C. gestroi *crude extract with a unique peptide match to a GH7. The enzyme source specifically cleaved internal glycosidic bonds of APTS-reducing-end-labeled-cellopentaose (arrows), releasing mainly cellotriose as hydrolysis product. **(B) **Complete hydrolysis of APTS-reducing-end-labeled-mannohexaose by IEX1-26, a positively screened FPLC fraction derived from *C. gestroi *crude extract with a unique peptide match to a GH2. The enzyme source specifically cleaved internal glycosidic bonds of mannohexaose (arrows), releasing mainly mannobiose and mannotriose as final product. **(C) **SDS-PAGE analysis of fractions IEX4-37 (lane1) and IEX1-26 (lane 2). A unique band of 50 kDa was observed after Coomassie blue staining for IEX4-37 (lane 1), and bands of about 80 kDa were observed for IEX1-26 after silver staining. (M) Protein Ladder. APTS: 8-aminopyreno-1,3,6-trisulfonic acid; FPLC: fast performance liquid chromatography; GH: glycoside hydrolases; IEX: ion-exchange.

Hydrolysis of *p*-nitrophenyl-carbohydrate (*p*NP) substrates had its most significant result against *p*NP-glucopyranoside (*p*NP-G), followed by *p*NP-cellobioside (*p*NP-C) and *p*NP-mannoside (*p*NP-M) (Figure 1C). For all *p*NP derivatives evaluated in this study, the optimal pH of operation occurred at pH 6 (Figure 1C). However, a drastic drop in the enzyme activity was observed at pH 7, of about 80%. *p*NP-α-L-arabinofuranoside (*p*NP-A) was barely hydrolyzed by the crude enzyme extract and no action was detected on *p*NP-xylopyranoside (*p*NP-X) in this study.

### Metaproteomic analysis

A metaproteomic analysis from the whole body of the lower termite *C. gestroi *was carried out. Our strategy allowed the identification of a hundred positive fractions that degrade at least one of the carbohydrates listed above. Following the functional screening, the positive fractions were subjected to trypsin digestion and ultra performance liquid chromatography (UPLC) followed by tandem mass spectrometry analysis. Since there is very little genomic sequence data available for *C. gestroi*, the LC-MS/MS spectra were analyzed by Mascot Ions-Search Software (Matrix Science, London, GB, UK)for protein identification using a database containing all non-redundant proteins derived from the CAZy website [20], a specialized database (up to 67,000 protein sequences) that describes the families of structurally-related catalytic and carbohydrate-binding modules (or functional domains) of enzymes that degrade, modify or create glycosidic bonds. After spectra acquisition, using a stringent cut-off (cut-off > 27; significant at *P *< 0.05) allowed the explicit assignment of 121 unique peptides as GHs and CBMs (Additional File 1).

Our proteomic approach successfully identified a large number of polypeptides in the *C. gestroi *digestome. A total of 55 different protein matches were identified and classified in 29 CAZy families (Figure 2). The sequence and distribution of the peptides identified through proteomic analysis, as well as their categorization in different GH and CBM families, are described in detail in Additional File 1. Using a confidence criterion of two or more unique peptides of different peptide sequences [21], three protein matches for GH members were identified. Potential cellulolytic enzymes were identified using this criteria, such as the match to probable β-glucosidase (GH3) from *Dictyoglomus turgidum*, two GH7 protein matches, one derived from *Pleurotus sp*. and another from the uncultivated symbiotic protist *Hodotermopsis sjoestedti*, as well matches to GH9, from *Reticulitermes flavipes *and *C. formosanus*, which are endogenous cellulases from termites [19,22-25]. Two protein matches with high confidence were assigned to CBM families 6 and 27, derived from *Salinispora arenicola *and *Thermotoga sp*., respectively (Additional File 1). CBM6 is often coupled to a GH16 catalytic domain [26], which has been described as acting against substrates that posses β-1,3 linkages such as lichenan, laminarin and curdlan. CBM27 is a protein domain that binds to mannose polymers, and is usually tied to GH26, β-mannosidase or β-xylosidase, forming a multidomain protein [20,27].

Based on the total number of peptides identified, the majority of components found in the *C. gestroi *digestome were cellulose degrading enzymes, including β-glucosidases (GH1 and GH3), which accounted for up to 10% of the protein matches, followed by GH5, GH7 and GH9 families, which made up 22% of the total peptides. These are enzymes that preferentially hydrolyze internal β-1-4 glycosidic bonds. Protein assignments for GH16 (3%), probable endo-β-1-3-glucanase, were also present in the termite extract (Figure 2). Xylanolytic enzymes, members of GH10 and GH11 families, also counted as an important component, comprising up to 6% of the total peptides identified. Three peptides with matches for family GH43, with known activities including β-xylosidase and α-L-arabinofuranosidase, were found in our analysis. Potential mannan-hydrolytic enzymes, members from families GH2 and GH38 (three peptides identified), as well as polygalacturonase(GH28), beta-galactosidase (GH42), fucosidases (GH29) and fructofuranosidases (GH32) were identified (one peptide match of each, respectively). A remarkable number of peptides with matches for starch-degrading and branching enzymes were found; matches for families GH4, GH13, GH31, GH57, GH63, GH65, GH85 and GH94 counted up to 22% of the total peptides matches. Protein matches for putative lysozymes were also found and GH25 and GH28 members, one peptide for each family, were identified. About 3% of the peptides matches found (two distinct protein matches) were identified as chitinases, based on GH18 classification.

The enzyme hydrolytic activity verified on chromatographic fractions selected for mass spectrometry analysis corroborated with their protein identification by LC-MS/MS, thereby validating the identification of proteins that have a unique peptide sequence found in our proteomic analysis (Additional File 1). Cellulolytic protein matches to GH1, GH3, GH5, GH7 and GH9 were mostly derived from the chromatographic fraction active on hydrolyzing CMC and lichenin. Among the peptides found with low confidence criteria, which were confirmed by the expected enzymatic hydrolysis, were members of families GH10, GH11 and GH43 (Additional File 1). Those were identified by their hydrolytic activity on xylan. The unique peptide match for GH16 was derived from a positive fraction with activity against lichenin, and another unique match for GH2 was derived from mannan degrading fractions.

With the aim of further validating our proteomic results, we analyzed a couple of ion-exchange (IEX) fractions through SDS-PAGE and ran a carbohydrate degradation profile by capillary zone electrophoresis. As shown in Figure 3A, the analysis by capillary electrophoresis of cellopentaose degradation by the IEX4-37 fraction, with just one peptide match identified as GH7, showed the derived hydrolysis products as being cellobiose and cellotriose. The enzyme source specifically cleaved internal glycosidic bonds of 8-aminopyreno-1,3,6-trisulfonic acid (APTS)-reducing-end-labeled-cellopentaose (Figure 3A), which was the expected mode of action for a GH7 type endoglucanase. The SDS-PAGE analyses for fraction IEX4-37 reveled one unique band around 53 kDa (Figure 3C), the predicted molecular weight for this protein match as suggested by the Mascot ion search. The purified fraction IEX1-26, which was identified as a single peptide match for the GH2 family, was able to hydrolyze mannohexaose, producing mannotriose and mannobiose, as expected for typical endo-β-1,4-mannanase hydrolysis (Figure 3B). The SDS-PAGE for IEX1-26 reveled protein bands of about 80 kDa (Figure 3C), compatible for mannanases derived from fungi and bacteria [27,28].

## Discussion

The molecular pathways of biomass conversion by termites have long intrigued researchers of both basic and applied biosciences. Here, we described the first global study of the enzymatic repertoire involved in plant polysaccharide degradation by the lower termite, *C. gestroi*, a main urban pest occurring in Brazil. The goal of our work was to expand the knowledge on plant biomass degradation by termites through culture-independent approaches, based on biochemical and global proteome characterization of termite cell extracts [7,29].

The study herein shows a comprehensive functional characterization of the crude body extracts from *C. gestroi*. We challenged the ability of the crude termite extract to degrade a wide collection of substrates, including natural polysaccharides and *p*NP derivatives. The *C. gestroi *digestome managed to break down all kinds of natural polysaccharides, highlighting the biotechnological potential for the discovery of new enzymatic components from termites [11,12,14,30,31]. Our data, evidencing that cell extracts from *C. gestroi *contained all enzymatic activities assayed, such as CMCase, β-glucosidases, endoglucanases, xylanases, manananases, pectinases and amylases, is in agreement with previous reports for various higher and lower termites [32-39].

The capacity of the termite to hydrolyze such a variety of glycoside bonds is a consequence of endogenous enzymes and lignocellulolytic microbiota living in the termite gut. Cellulose and xylan utilization has been described before in the digestive track of a number of lower and higher termite species [25,33,36,39,40]. Likewise, a number of cellulose and xylan degrading enzymes were previously purified and/or functionally characterized from termites, such as endogenous endoglucanases, classified as GH9, from *C. formosanus*, *R. speratus *and *Nasutitermes *spp. [7,23,38,41], β-glucosidases (GH1) from *R. flavipes *[42] and *Neotermes koshunensis *[43,44], an endo-β-glucanase (GH7) from a *symbiotic protozoa in the hindgut of Coptotermes sp *[45,46] and an endo-β-xylanase (GH 11) from *C. formosanus *[40] and from *Nasutitermes *spp. [47].

Meanwhile, there is a paucity of studies describing enzymatic hydrolysis of mannan polysaccharides by termites or symbiotic microbiota [34]. The detection of enzymatic hydrolysis of polysaccharides, such as laminarin, lichenan, xyloglucan, arabinogalactan, arabinan and pectin, by crude termite extracts, to the best of our knowledge, have not yet been reported. The detection of these enzymatic activities should be expected, based on the previous isolation of hemicellulolytic-degrading bacteria and yeast from the termite gut, which has already allowed the identification of enzymatic activities such as endo-xylanase, β-xylosidase, α-L-arabinofuranosidase, β-galactanase and β-galactosidases [35].

The capability to hydrolyze *p*NP-G and *p*NP-C indicated the occurrence of β-glucosidases and cellobiosidases, which were previously described in the genera *Nasutitermes *and *Neotermes *[36,43,44]. Enzymatic degradation of *p*NP-X and *p*NP-A, which were described for gut bacteria and yeast, have not been observed in *Neotermes jouteli *cell extracts [35], corroborating the data in the current study. However, β-xylosidase activity was identified in the midgut and hindgut portions of *R. speratus *[36]. It is important to underline that there is a correlation between termite diet and the intestinal microbial population suitable for digestion. Therefore, it is expected that some enzymatic components may appear induced or repressed, which could explain some of the variation in enzymatic activities observed in our study [48].

The great efficiency of plant biomass degradation by termites is a consequence of the enzymatic action occurring throughout the termite gut, carried out by enzymes both endogenous and derived from symbiotic microbiota. The results for enzymatic assays carried out in different pHs drew our attention to a slight variation in enzyme extract effectiveness. Except for the arabinoxylan and synthetic substrate activity assays, enzyme optimal activity range was between pH 4 and pH 8. As described by others, a strong variation in pH occurs between gut compartments, consistent with the hydrogen potential profile of the termite gut [6], varying from very acidic (pH 3) to very basic (pH 12). However, the average pH in the gut is around pH 5 to pH 6, which correlated to the optimal results described herein, as has also been described by previous reports [34].

The proteomic strategy presented herein was successful, based on the number of glycoside hydrolase family members identified through our efforts. We identified 55 different GH enzymes and two CBM proteins, within 29 different CAZy families (Figure 2). Our data are similar to a previous report from *N. corniger*, which identified 48 proteins within 22 CAZy and Pfams [17], whereas Warnecke *et al*. [14] reported 13 CAZY proteins. Using a high confidence criterion, we identified protein matches for GH3, GH7 (two matches), GH9 and two CBMs from families 6 and 27. Previous proteomic efforts from Burnum *et al*. [17] retrieved members of six CAZy families (eight different protein targets), that passed filtering criterion of more than two unique peptide identifications. Several protein hits for polysaccharidases were found in our proteomic study with low significance, which may be justified by enzymes that were insoluble and/or remained firmly bound to lignocellulose or the unavailability of a representative genome, coupled by dealing with a microenvironment where proteins may be under-represented [49].

The proteomic study presented herein increased the range of known enzymes present in the lower termite digestome. We have found protein identifications for cellulose degrading enzymes (GH1, GH3, GH5, GH7 and GH16), xylan (GH10, GH11 and GH43) and mannan (GH2 and GH38) degrading components, pectinases (GH28 and GH29) and β-galactosidases (GH42), as well as amylases (GH13, GH31 and GH57) and chitinases (GH18 and GH85). Fewer than half these components were previously reported in the proteome of *N. corniger*; the exceptions being GH2, GH7, GH10, GH11, GH16, GH18, GH28, GH29, GH31, GH38 and GH57 [14,17]. Metagenome sequencing of the hindgut of *N. corniger *have confirmed the coding genes for all protein hits identified by our study, but not for GH7, GH10 and GH29, which are virtually absent in higher termite metagenomes [14,17]. On the other hand, confirming our proteomic data, carbohydrate-active transcripts for GH16 have been reported for termite symbionts and beetles [12,50,51] and GH7 have been retrieved in fungi and termite symbionts; GH 9 from *C. gestroi *was retrieved from the expressed sequence tag library, and GH2, GH3, GH11, GH18 and GH38 were represented in the host and symbiont sequence pools from *Reticulitermes *spp.

Our strategy not only enabled us to push forward the identification of glycoside hydrolases, but also validated our results for protein identification with both high and low confidence criteria (identification of only one unique peptide). The protein matches with low confidence, such as GH10, GH11 and GH 43, were derived from chromatographic fractions that degraded xylan, likewise proteins matching GH2, GH38 and CBM27 corresponded to fractions with hydrolytic activity on mannan. GH16 was identified from chromatographic fractions with high hydrolytic activity on lichenan. Moreover, the partial purification of two enzymes with low confidence followed by characterization using specific APTS-labeled oligosaccharides, made two low confidence protein matches for GH2 and GH7 more reliable. Collectively, our findings can offer a first glimpse of the GH range existing in the lower termite digestome, as well as help to suggest target genes for cloning and heterologous expression in future termite-based biomass conversion strategies. The use of robust LC-MS/MS analyzers and deep sequencing in a third generation sequencer could generate large data sets for termite digestome analysis, and further studies may be required to reveal additional components, such as lignin degrading proteins.

## Conclusions

The biochemical characterization analysis presented herein showed, for the first time, that the lower termite, *C. gestroi*, possess the ability to cleave almost all the glycosidic bonds contained in 18 polysaccharide substrates tested at a pH range of pH 3 to pH 8. Furthermore, the metaproteomic approach reveled the repertoire of glycoside hydrolase families involved in biomass conversion in the *C. gestroi *digestome, such as cellulase members from GH1, GH3, GH5, GH7, GH9 and CBM 6, hemicellulases GH2, GH10, GH11, GH16, GH43 and CBM27 and pectinases GH28 and GH29. We expect that our findings will provide the biochemical and molecular basis for further studies on termite biology and digestome physiology, from which a number of important applied research topics can be generated, such as the development of novel enzymatic routes for use in biomass-to-bioethanol applications.

## Methods

### Termites

Workers of *C. gestroi *were collected from field colonies with traps of corrugated cardboard and from incipient colonies reared in the Termite Laboratory of the Biology Department, UNESP, Rio Claro, São Paulo, Brazil (22823'S, 47831'W). Termites were kept at room temperature with nest materials and worker termites manually selected and utilized for all experiments.

### Crude enzyme extract preparation

Total protein extractions for biochemical characterization were prepared from 50 whole termite bodies. They were homogenized using Potter-Elvehjem Tissue Grinder (Wheaton, Millville, NJ, USA) with 1 mL of 50 mM McIlvine [52] buffer extraction at pH 5.0. After extraction, the mixture was centrifuged at 20,100 g for 20 minutes at 4°C. The supernatant was collected and is here referred as crude enzyme extract. Protease inhibitor cocktail (Anresco - Solon, OH, USA) was added to the crude extract produced (1 μL/mL). The concentration of the protein in crude extract was determined by the Bradford method [53] All experimental procedures were performed on ice.

### Enzymatic assays

The assays for biochemical characterization were conducted with 10 μL of crude enzyme extract (3.2 μg/μL) incubated with 40 μL of 50 mM McIlvine buffer (pH 4 to pH 8) and 50 μL of 0.5% specific substrate (in water), in 96-well PCR plates, in triplicate, at 37°C, for 20 minutes to 60 minutes, depending on the substrate. Substrates were purchased from Megazyme (Wicklow, Ireland) and Sigma Aldrich (St, Louis, MO, USA). Enzymatic assays were stopped by the addition of 100 μL of dinitrosalicylic acid [54] and heated at 99°C for 5 minutes. The measurement of color change was performed at 550 nm using a microplate reader (InfiniteM200 Tecan - Männedorf, Switzerland). The enzymatic assays with *p*NP were performed as follows: 10 μL of crude extracts were incubated with 10 μL of 5 mM *p*NP substrates, 30 μL of 50 mM McIlvine buffer (pH 4 to pH 8) and 10 μL MilliQ-Water (Millipore - Billerica, MA, USA). Assays were stopped by the addition of 100 μL of 1 M sodium carbonate. The measurement of color change was performed at 400 nm using a microplate reader. The enzymatic assays were done in triplicate. Glucose and *p*NP were used for the standard curve construction. The enzymatic activity assays results were expressed in terms of mM of glucose equivalents produced

### Chromatographic separation steps through ion-exchange and gel filtration

The metaproteomic analysis on the whole body of *C. gestroi *was carried out to reveal polysaccharidases contained in the termite digestome. To reduce the complexity of the sample, the termite crude extract was submitted to chromatographic separation followed by enzymatic activity screening of purified fractions. Four types of polysaccharides were used as substrates for enzymatic activity assays, namely CMC, lichenan, mannan and xylan.

Prior to FPLC-LC-MS/MS analysis, about 500 termites were used for crude enzyme extract preparation, as described above, using 100 mM phosphate-potassium buffer (PPB), pH 6.2. The ion-exchange (IEX) purification chromatography was carried out using a Resource Q anionic exchange column on an AKTA Purifier System (both GE Healthcare Life Sciences - Piscataway, NJ, USA). Initially, 1.5 mL of crude extract was cleared on a Millipore filter of 0.45 μm and then 1 mL was applied onto the Resource-Q column, equilibrated with 100 mM PPB, pH 6.2. The termite crude extract was fractionated through a linear gradient (100 mL) of 0 to 1 M sodium chloride in 100 mM PPB, pH 6.2, at a flow rate of 1.0 mL/min and collecting 2-mL fractions. IEX purifications were repeated five times, generating 250 eluted fractions. These fractions were submitted to enzymatic assays against the polysaccharide substrates at room temperature for 24 hours. Fractions with positive enzymatic activity were selected for LC-MS/MS analysis.

Fractions derived from IEX positively selected for lichenanases, xylanases, CMCase and mannanases activity screenings were applied onto Hi-Load 16/60 Superdex 75 - Gel Filtration Column (GE Healthcare), equilibrated in 100 mM PPB, and eluted in 100 mM PPB, pH 6.2 containing 0.5 M sodium chloride, at a flow rate of 0.1 mL/min with 2-mL fractions. Fractions of gel filtration chromatography with positive activity against the substrates described above were selected for LC-MS/MS analysis.

### Mass spectrometer analysis (LC-MS/MS)

About 500 μL of eluted fractions from chromatography steps were precipitated with ice cold acetone (Merck - Darmstadt, Germany) (1:2, v:v), then incubated at -20°C for 2 hours, followed by centrifugation at 4°C. The supernatant was recovered and the pellet was dried with a Speed-Vac (Eppendorf - Hauppauge, NY, USA) for 30 minutes and then resuspended into 100 μL of 50 mM ammonium bicarbonate. Proteins were reduced, alkylated and the reduction step repeated. Proteins were then digested with 1 μg trypsin (Porcine Prep Grade Sequence, Promega - Madison, WI, USA), in 1 mM calcium chloride) at 37°C overnight. The reaction was stopped by adding 0.1% formic acid (Merck) and dried in the Speed-Vac. Each sample was resuspended in 12 μL formic acid, 0.1%, and an aliquot (4.5 μL) of the resulting peptide mixture was separated by C18 (100 μm × 100 mm) RP-nanoUPLC (nanoAcquity, Waters) coupled with a Q-Tof Ultima mass spectrometer (Waters) with a nano-electrospray source at a flow rate of 0.6 μL/min. The gradient was 2% to 90% acetonitrile in 0.1% formic acid over 60 minutes. The instrument was operated in the 'top three' mode, in which one MS spectrum is acquired followed by MS/MS of the top three most intense peaks detected. The spectra were acquired using the software MassLynx v.4.1 (Waters - Milford, MA, USA) and the raw data files were converted to a peak list format (mgf), without summing the scans, by the software Mascot Distiller v.2.3.2.0, 2009 (Matrix Science Ltd.). These were then searched against the CAZy Database (67,651 sequences; 33,940,339 residues [20]) using Mascot v.2.3.01 engine (Matrix Science Ltd.), with carbamidomethylation as the fixed modification, oxidation of methionine as a variable modification, one trypsin missed cleavage and a tolerance of 0.1 Da for both precursors and fragment ions. Only peptides with a minimum of five amino acid residues, with a significance threshold of *P *< 0.05 (Mascot-based score) were considered as a product of peptide cleavage. The peptide was considered as unique when it differed in at least one amino acid residue and covalently modified peptide, including N- or C-terminal elongations (that is, missed cleavages). Different charging states of the same peptide and modifications were not counted as unique.

### Capillary electrophoresis

Cellopentaose and mannohexaose (Megazyme) were derivatized with APTS by reductive amination, as described by dos Santos *et al*. [55]. Enzymatic reactions were performed as described for standard enzymatic activity, except that 1 pM of APTS-labeled specific oligosaccharides were used as substrates. Capillary zone electrophoresis was performed on a P/ACE MQD (Beckman Coulter - Indianapolis, IN, USA) with laser-induced fluorescence detection. A fused silica capillary (TSP050375, Polymicro Technologies - Phoenix, AZ, USA) of internal diameter 50 mM and length 31 cm was used as separation column for the oligosaccharides. Samples were injected by the application of 0.5 psi for 0.5 seconds. Electrophoresis conditions were 10 kV/70-100 mA with the cathode at the inlet, 0.1 M sodium phosphate, pH 2.5, as a running buffer, and a controlled temperature of 20°C. The capillary was rinsed with 1 M sodium hydroxide followed by running buffer to prevent carryovers. APTS-labeled oligomers were excited at 488 nm and emission was collected through a 520-nm band pass filter. Because of the small volumes of capillary electrophoresis combined with small variations in buffer strength, retention times varied slightly when comparing separate electrophoresis runs. The combined information, obtained from the electrophoretic behavior and co-electrophoresis with oligosaccharides, was used for the identification of formed products.

## Abbreviations

APTS: 8-aminopyreno-1,3,6-trisulfonic acid; CBM: carbohydrate binding module: CMC: carboxymethyl cellulose; FPLC: fast performance liquid chromatography; GH: glycoside hydrolases; GT: glycoside transferases; IEX: ion-exchange; LC-MS/MS: liquid chromatography with tandem mass spectrometry; PCR: polymerase chain reaction; *p*NP: *p*-nitrophenyl-carbohydrate; PPB: phosphate-potassium buffer; UPLC: ultra performance liquid chromatography.

## Competing interests

The authors declare that they have no competing interests.

## Authors' contributions

JPLFC performed the experimental design, biochemical assays, molecular biology experiments, chromatography purification steps, mass spectrometer sample preparation, data analyses and drafted the manuscript. FCL performed the termite cultivation and biochemical assays. AMCL performed the termite cultivation. TMA managed the carbohydrate capillary electrophoresis analysis, helped in the interpretation of data and drafted the manuscript. DAR and FB performed the mass spectrometer sample preparation and its protocol establishment. FFC helped with the experimental design. MFC performed the bioinformatics analyses. AFPL managed the mass spectrometer analyzer, helped in the data analyses and drafted the manuscript. GAGP helped with the experimental design. FMS conceived, designed and coordinated the study, performed the data analyses and wrote the manuscript. All authors suggested changes to the manuscript, commented on preliminary versions of the paper, and approved the final text.

## Supplementary Material

Additional file 1 **Table 1. Glycoside hydrolases and protein carbohydrate-binding modules identified from *C. Gestroi*.** Prior to LC-MS/MS, the termite crude extract was submitted to chromatographic separation steps through ion-exchange (IEX) and gel filtration (GF), followed by enzymatic activity screening of purified fractions. The results of enzymatic activity assays of the chromatographic fractions were expressed in terms of mM of glucose equivalents produced, against the following substrates: L = lichenan; M = mannan; C = carboxymethyl cellulose; X = xylan. In cases that different fractions identified the same peptide, only the higher enzymatic activity observed is reported, which is the first "IEX#" fraction listed. Click here for file
