# Supplementary material for: Functional characterization and target discovery of glycoside hydrolases from the digestome of the lower termite Coptotermes gestroi
Source: Biotechnol Biofuels. 2011 Nov 14;4:50. doi: 10.1186/1754-6834-4-50 (PMC3285041; doi:10.1186/1754-6834-4-50)
Supplement: Additional file 1 — Table 1. Glycoside hydrolases and protein carbohydrate-binding modules identified from C. Gestroi. Prior to LC-MS/MS, the termite crude extract was submitted to chromatographic separation steps through ion-exchange (IEX) and gel filtration (GF), followed by enzymatic activity screening of purified fractions. The results of enzymatic activity assays of the chromatographic fractions were expressed in terms of mM of glucose equivalents produced, against the following substrates: L = lichenan; M = mannan; C = carboxymethyl cellulose; X = xylan. In cases that different fractions identified the same peptide, only the higher enzymatic activity observed is reported, which is the first "IEX#" fraction listed. [file 1754-6834-4-50-S1.DOC]

***Table 1.*** Glycoside hydrolases and protein carbohydrate-binding modules identified from *C. gestroi*

***Table 1.*** continued

***Table 1.*** continued
